# Supplementary material for: Targeted Mutagenesis of the Female-Suppressor SyGI Gene in Tetraploid Kiwifruit by CRISPR/CAS9
Source: Plants (Basel). 2020 Dec 30;10(1):62. doi: 10.3390/plants10010062 (PMC7823651; doi:10.3390/plants10010062)
Supplement: Supplementary file 1 [file plants-10-00062-s001.pdf]

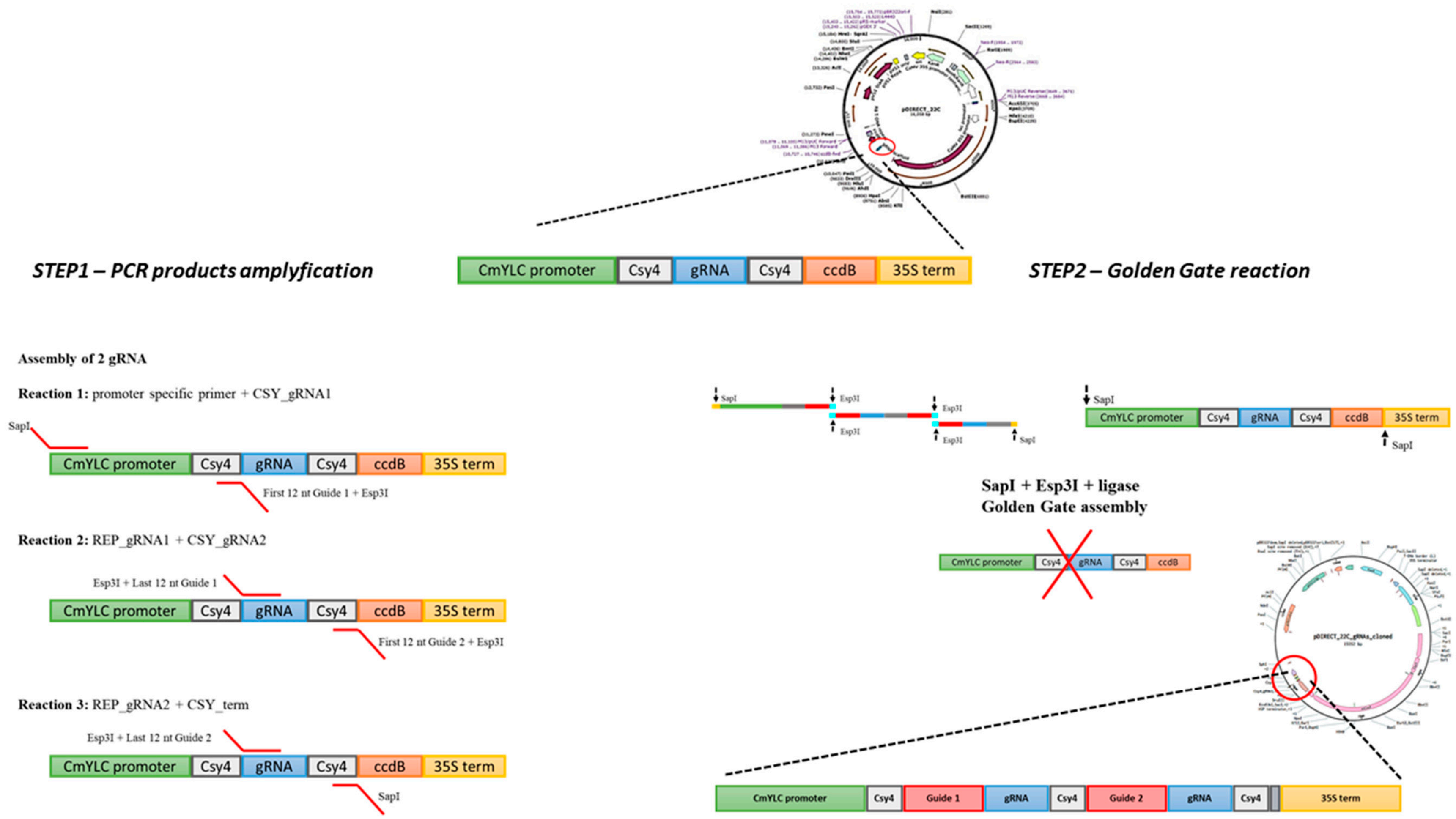

Figure S1. Golden gate assembly of p*DIRECT22C*:gRNAs vector. The p*DIRECT22C* vector serves both as the cloning backbone and as the template for PCR amplification of individual parts. PCR products are assembled into the vector using two distinct type of enzymes: *Sap*I and *Esp*3I. Clevage by *Sap*I will release the *ccdB* cassette which will be replaced by the final p*DIRECT22C*:gRNA vector cassette containing the two cloned sgRNAs. More details are reported by Čermák *et al.*, [21].

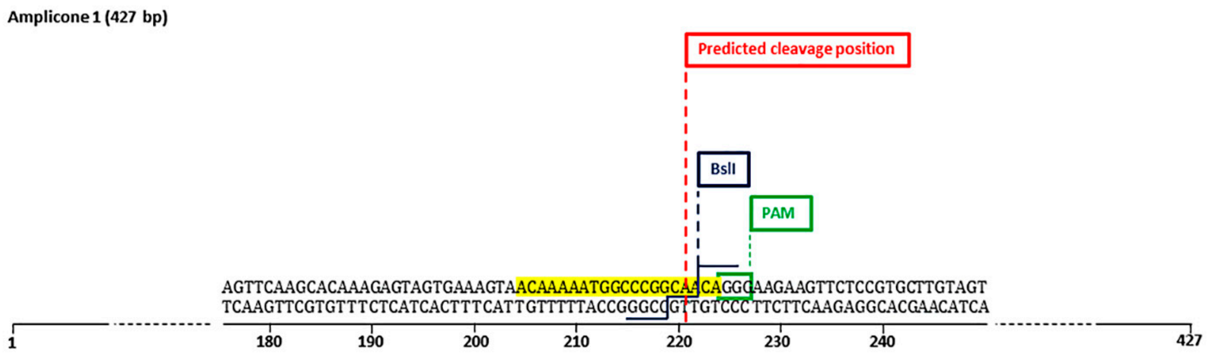

Figure S2. Restriction site for the *Bsl*I restriction enzyme within the predicted cleavage site in the sgRNA1 *locus*. The sgRNA1 is highlighted in yellow and its relative PAM sequence is underlined in green. The predicted cleavage site is reported in red and *Bsl*I restriction site is underline in blue. This site was exploited to set up a screening assay of the transformed plants to verify the success of the editing. The amplicon used in the restriction assay is 427-bp in length and displays two *Bsl*I restriction sites within the *wild type* sequence at position 222 nt (the predicted cleavage site defined by sgRNA1) and 320 nt, producing three fragments of 221, 98 and 108 bp after enzyme digestion. In the case of an edited plant, the cutting site at position 222 is supposed to be modified by the editing, providing a different digestion pattern (two fragments of 320 and 108 bp).

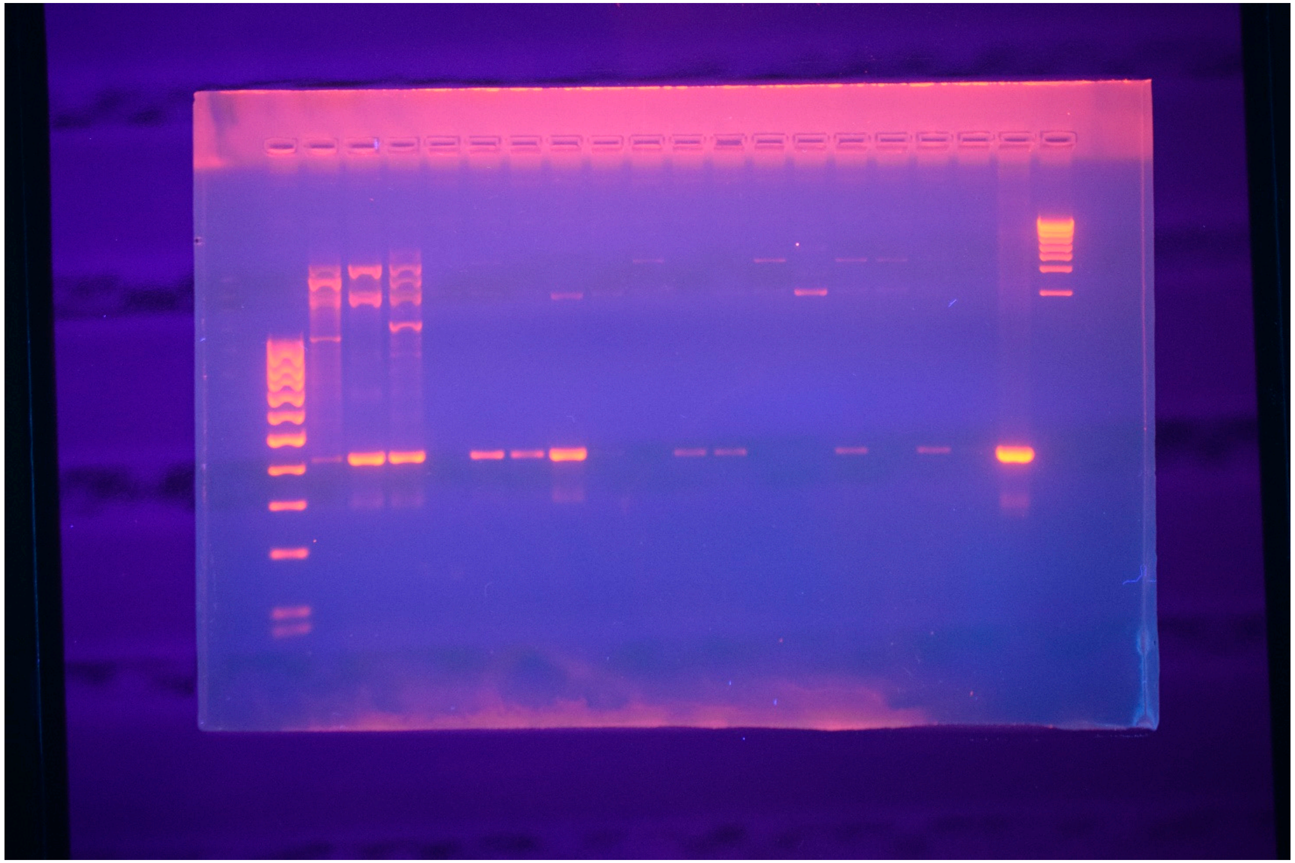

Figure S3. PCR detection of p*DIRECT22C*:gRNAs vector integration within the genome of the 11 putative transgenic shoots obtained for the male genotype A0134.41 and 4 putative transgenic shoots obtained for the Ac174.46 cultivar. The primer pair TC320/TC089R amplifies the p*DIRECT22C*:gRNA vector cassette where the two guideRNAs were cloned, producing a 418 bp fragment. GeneRulerTM 100 bp DNA Ladder (Thermo Fisher) was used for sizing of the PCR products in the range of 100 bp to 1,000 bp on agarose gel. The last two samples represent the negative control (NC, Milli-Q water) and the positive control (p*DIRECT22C*:gRNAs vector) respectively. The illustrative picture (Fig.3 in the manuscript) has been cut deleting the upper part with unspecific DNA amplifications. The color balance was applied to every pixel in the image. The changes do not alter the information illustrated in the original figure. Plant line code of each putative transgenic shoot and the ladder name were manually added in the Figure 3 (in the manuscript).

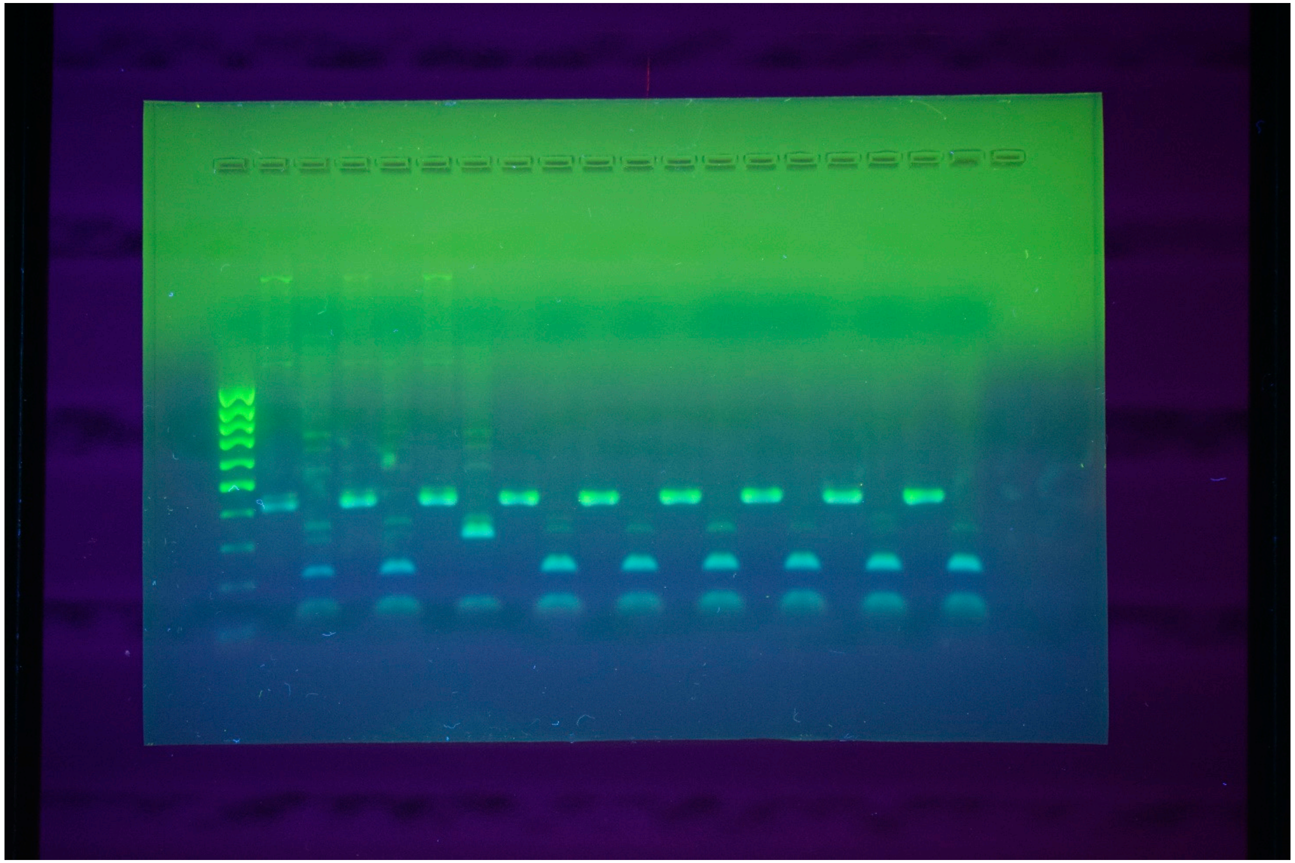

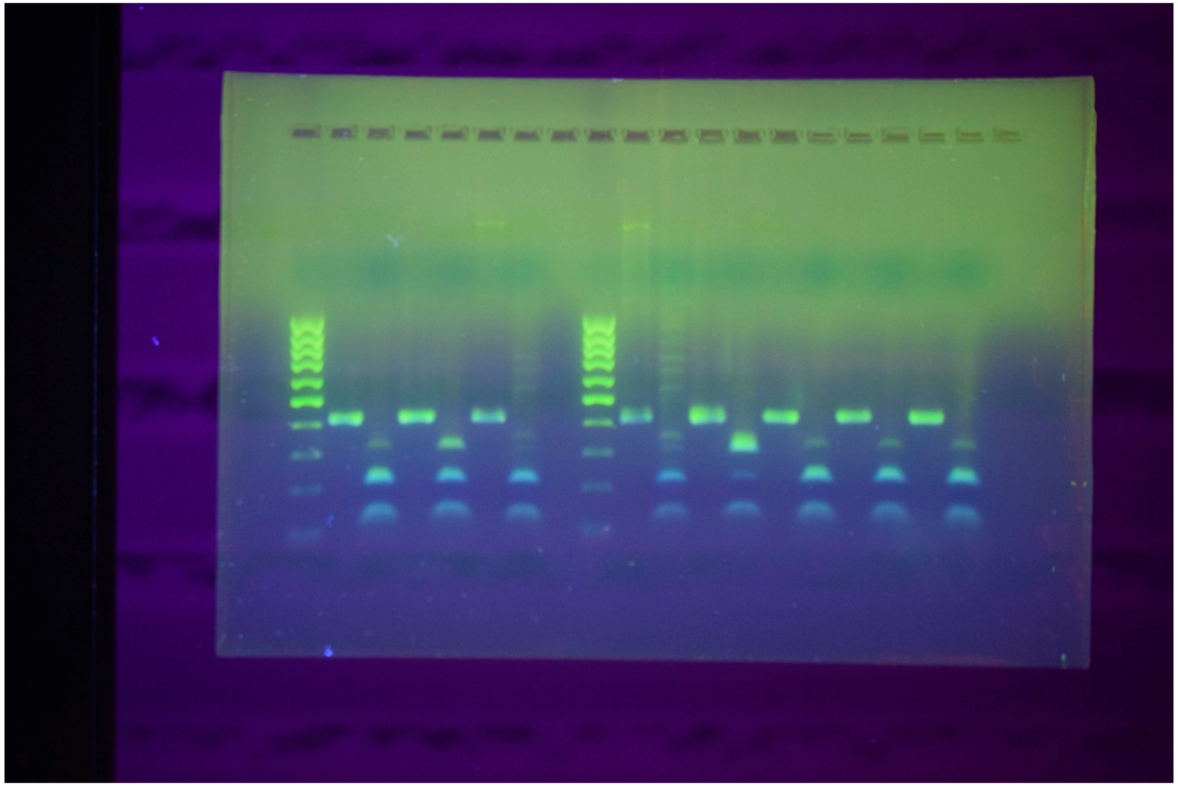

Figure S4. Agarose gel electrophoresis of the sgRNA1 target sequence PCR indigested product and their *Bsl*I digestion. The male genotype A0134.41 *wild-type* (WT), plus the 11 putative transgenic shoots obtained from this male genotype, are displayed in the the first gel and in the first three rows of the second ones. The male genotype Ac174.46 *wild-type* (WT) plus the 4 putative transgenic shoots obtained from this male genotype are displayed in the second gel. GeneRulerTM 100 bp DNA Ladder (Thermo Fisher) was used for sizing the PCR products in the range of 100 bp to 1,000 bp on agarose gel. The plant line code of each sample is reported. The amplicon of the sgRNA1 target assay is 427-bp in length and his *Bsl*I digestion produces three fragments of 221, 98 and 108 bp (the latter two bands appear as one). PCR amplification of some lines, i.e A0134.41, *wild-type* (WT), A0134.41_L5 and Ac174.46 *wild-type* (WT), co-amplifies a second sequence which results in the presence of two extra-bands in the restriction pattern. Thirteen of these plants exhibited a restriction pattern compatible with the *wild-type*, showing a fragment of 221 bp. The same assay on an edited sequence provides a different digestion pattern, producing two fragments (320 bp and 108 bp). Image for illustrative purpose. The color balance was applied to every pixel in the image. The changes do not alter the information illustrated in the original figure. The plant line code of each putative transgenic shoot and the ladder name were manually added in the Fig. 4 (in the manuscript).

Table S1. Primer sequences used in this study.

Primer Name	Sequences	Note	Target
**Primer pairs for cloning**			
**promoter_CmYLCV**	TGCTCTTCGCGCTGGCAGACATACTGTCCCAC	PCR1	pDIRECT22C vector
**CSY_gRNA1**	TCGTCTCCGGCCATTTTTGTCTGCCTATACGGCAGTGAAC		
**REP_gRNA1**	TCGTCTCAGGCCCGGCAACAGTTTTAGAGCTAGAAATAGC	PCR2	pDIRECT22C vector
**CSY_gRNA2**	TCGTCTCCAACGGCTTCACACTGCCTATACGGCAGTGAAC		
**REP_gRNA2**	TCGTCTCACGTTGACTGCTGGTTTTAGAGCTAGAAATAGC	PCR3	pDIRECT22C vector
**CSY_term**	TGCTCTTCTGACCTGCCTATACGGCAGTGAAC		
**Primer pair for colony screening §**			
**TC320**	CTAGAAGTAGTCAAGGCGGC		T-DNA in pDIRECT22C vector
**TC089R**	GGAACCCTAATTCCCTTATCTGG		
**Primer pairs for mutation detection**			
**SyGI-amplicon1-F**	CGCTTTCATGGGATTAATGCTAG		genomic DNA from kanamycin-resistant
**SyGI-amplicon1-R**	GGAGCTAACCTTGGGACCAT		adventitious buds
**SyGI-amplicon2-F**	ATTCGTCCACCAACTCCCTT		genomic DNA from kanamycin-resistant
**SyGI-amplicon2-R**	TCCAACCTTTCCAAAACAGTTCA		adventitious buds

§ The same primer pair has been used to check T-DNA integration into genomic DNA.

Table S2. Composition of media used in this study.

Medium Type	Composition
**A. tumefaciens Re-suspension medium§**	▪ MS half strength [37]<br>▪ Vitamins [49]<br>▪ Supplemented with 2% sucrose and 100 μM acetosyringone
**Co-cultivation medium§**	▪ MS basal medium [37]<br>▪ Vitamins [49]<br>▪ Supplemented with 2mg/L of zeatin, 1,5 mg/L of 6-benzylaminopurine, 0,1 mg/L of NAA, 50 μM acetosyringone, 3 % sucrose and 0,25% of phytagel
**Re-generation and selection medium§**	▪ MS basal medium [37]<br>▪ Vitamins [49]<br>▪ Supplemented with 2mg/L zeatin, 1,5 mg/L 6-benzylaminopurine, 0,1 mg/L NAA, 150 mg/L kanamycin, 300 mg/L Cefotaxime, 3 % sucrose and 0,25% phytagel
**Shoot elongation medium§**	▪ MS basal medium [37]<br>▪ Vitamins [49]<br>▪ Supplemented with 0,1 mg/L IBA, 50 mg/L kanamycin, 300 mg/L Cefotaxime, 3 % sucrose and 7 g/L Plant agar (B&V Parma)
**Shoot induction and maintenance medium**	▪ MS basal medium [37]<br>▪ Vitamins [49]<br>▪ Supplemented with 0,2 mg/L of IAA, 0,5 ,g/L of 6-benzylaminopurine, 2,5 % sucrose, 0,5% glucose and 7 g/L Plant agar (B&V Parma)

§ Medium composition is from Wang *et al*., 2007 [47].
